# Belowground fungal community diversity and composition associated with Norway spruce along an altitudinal gradient

**DOI:** 10.1371/journal.pone.0208493

**Published:** 2018-12-05

**Authors:** Max E. Schön, Kay Nieselt, Sigisfredo Garnica

**Affiliations:** 1 University of Tübingen, Institute of Evolution and Ecology, Plant Evolutionary Ecology, Tübingen, Germany; 2 University of Tübingen, Center for Bioinformatics (ZBIT), Integrative Transcriptomics, Tübingen, Germany; 3 Universidad Austral de Chile, Instituto de Bioquímica y Microbiología, Casilla, Isla Teja, Valdivia, Chile; Friedrich Schiller University, GERMANY

## Abstract

Altitudinal gradients provide valuable information about the effects of environmental variables on changes in species richness and composition as well as the distribution of below ground fungal communities. Since most knowledge in this respect has been gathered on aboveground communities, we focused our study towards the characterization of belowground fungal communities associated with two different ages of Norway spruce (*Picea abies*) trees along an altitudinal gradient. By sequencing the internal transcribed spacer (ITS) region on the Illumina platform, we investigated the fungal communities in a floristically and geologically relatively well explored forest on the slope of Mt. Iseler of the Bavarian Alps. From fine roots and rhizosphere of a total of 90 of Norway spruce trees from 18 plots we detected 1285 taxa, with a range of 167 to 506 (average 377) taxa per plot. Fungal taxa are distributed over 96 different orders belonging to the phyla *Ascomycota*, *Basidiomycota*, *Chrytridiomycota*, *Glomeromycota*, and *Mucoromycota*. Overall the *Agaricales* (438 taxa) and *Tremellales* (81 taxa) belonging to the *Basidiomycota* and the *Hypocreales* (65 spp.) and *Helotiales* (61 taxa) belonging to the *Ascomycota* represented the taxon richest orders. The evaluation of our multivariate generalized mixed models indicate that the altitude has a significant influence on the composition of the fungal communities (p < 0.003) and that tree age determines community diversity (*p* < 0.05). A total of 47 ecological guilds were detected, of which the ectomycorrhizal and saprophytic guilds were the most taxon-rich. Our ITS amplicon Illumina sequencing approach allowed us to characterize a high fungal community diversity that would not be possible to capture with fruiting body surveys alone. We conclude that it is an invaluable tool for diverse monitoring tasks and inventorying biodiversity, especially in the detection of microorganisms developing very ephemeral and/or inconspicuous fruiting bodies or lacking them all together. Results suggest that the altitude mainly influences the community composition, whereas fungal diversity becomes higher in mature/older trees. Finally, we demonstrate that novel techniques from bacterial microbiome analyses are also useful for studying fungal diversity and community structure in a DNA metabarcoding approach, but that incomplete reference sequence databases so far limit effective identification.

## Introduction

Mountain ecosystems offer a mosaic of habitats along an altitudinal gradient showing dramatic changes in abiotic and biotic conditions [1]. In general, altitudinal gradients are characterized by decreasing temperature with increasing altitude and its variation is accompanied with changes in soil properties and also precipitation, ultraviolet radiation and atmospheric pollution creating complex environmental changes [2]. Thus, altitudinal gradients can be considered as true “natural laboratories” because they permit us to investigate, within a relatively short geographic distance, environmental drivers affecting above- and belowground biota. For example, the impact of the changing altitude on species diversity [3], especially for plants and animals [4–7] has been studied for several years. Previous research indicated that these groups of macroorganisms follow at least three patterns: (i) diversity decreases monotonically with increasing elevation, (ii) diversity is high across lower elevations and lower at middle and high altitudes or (iii) diversity shows a hump-shaped relationship with a mid-elevational peak in richness [3,8,9]. In contrast, research investigating the effect of the altitude on the diversity of other living organisms such as soil microorganisms is still limited.

It has been hypothesized that fungal microorganisms follow more complex patterns of diversity compared to plants and animals and the implicated drivers are still poorly understood. So far, similar patterns as found for plants and animals have been reported for fungi, namely that the diversity is lower at lower elevation communities [10], decreases at high altitude [11–14] or peak at mid-altitude [15]. However, different patterns have also been reported, i.e., that the diversity increases at higher altitude [16] or does not change at all over elevation gradients [17–19]. In addition to the diversity, the community composition varies with elevation, as observed for communities in the beech (*Fagus* spp.) phyllosphere [20], from soil [21], associated with mycorrhizal plants [11,14,22,23] and of wood decomposing fungi [17]. Coince *et al*. [24] as well as Siles and Morgesin [19] found that the change in community composition was affected by edaphic and/or climatic variables rather than change in elevation. Matsuoka *et al*. [25] found that ectomycorrhizal (ECM) community composition can be explained by differences in both vegetation and abiotic factors.

**Fig 1:**
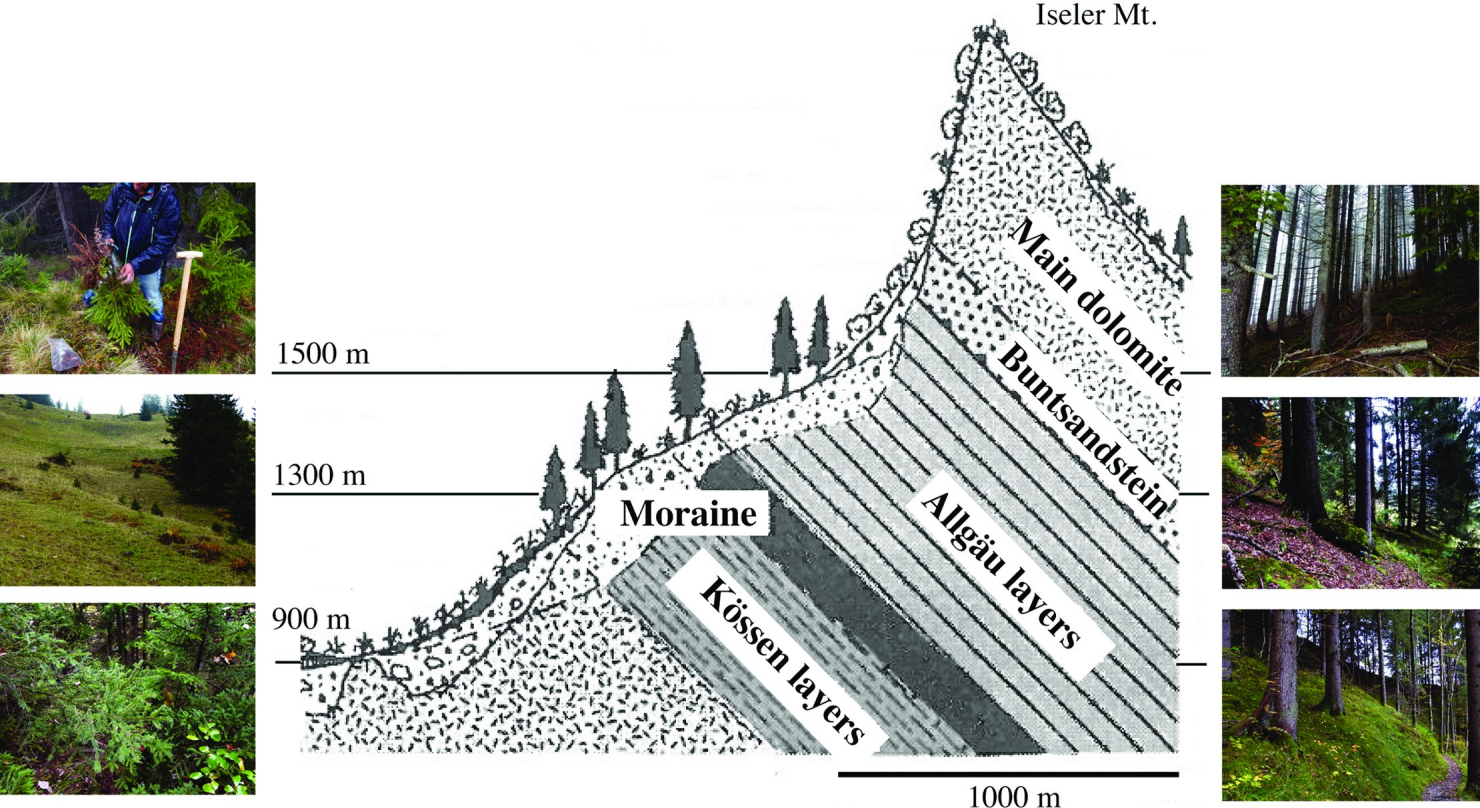
Overview of the study area comprising three sampling sites at three different altitudes along an altitudinal gradient from 900 to 1500 m a.s.l. Collection sites were located at I: 900 m, II: 1200 m and III:1500 m a.s.l in the Bavarian Alps (north side of Iseler Mountain, Germany), modified from Bräker [28]. At each location samples from old and young individuals of Norway spruce were sampled.

In this study, we investigated the belowground fungal communities along an altitudinal gradient associated with a single host [Norway spruce; *Picea abies* (L.) H. Karst.] and focused on the role of altitude and host age on diversity and composition. By focusing on plant monoculture, it is possible to analyse which role specific factors have on the fungal community diversity and composition independent of host vegetation change. In the Bavarian alps (Germany; Fig 1), the slopes of Mount Iseler (1878 m) include montane and subalpine ecosystems, which fall within the natural range of distribution of P. abies. However, the sampled plots represent rather secondary pure stands over 100 years old but some young trees are frequent at the forest margins. For these plots, there are detailed investigations on the local geology and soil chemistry as well as surveys on the aboveground fungal diversity that have been well documented by the last author of this paper over ten years [1,26,27].

Here we analyse the diversity and composition of belowground fungal communities associated with pure stands of Norway spruce trees of two different ages along an altitudinal gradient at elevations between 900 m and 1500 m above sea level (a.s.l) on the Mount Iseler. The main objectives for this study were (i) to assess the belowground fungal diversity and composition associated with Norway spruce along an altitudinal gradient in the Bavarian Alps, (ii) to directly compare diversity and composition of the fungal communities between stands of Norway spruce across different altitudes and ages, and (iii) to identify and ecologically characterize the most important fungal lineages and taxa.

In this work, belowground fungal taxa identifications were performed by comparing merged ITS sequences against annotated reference sequences using the tool MALT [29,30]. By applying state-of-the-art tools developed for large-scale microbiome analyses of bacteria, we made use of the significant advances made in this field over the last years [31–33]. With our study, we demonstrate that these tools are also expedient for fungal metabarcoding studies. In addition, using a multivariate generalized mixed model (mGLM), we study the putative effect of the variables altitude and host age on the belowground fungal communities and infer the functional groups (ecological guilds) present with FUNGuild [34].

## Materials and methods

### Sample collection

Sampling was conducted along an altitudinal gradient on the northern slope of Mt. Iseler in the Bavarian Alps near Oberjoch (Germany) (Fig 1). We collected at 900 m (sites O-I and Y-I), 1300 m (sites O-II and Y-II) and 1500 m (sites O-III and Y-III) altitude from old and young Norway spruce trees where no other ECM plants were growing nearby. From each altitude, randomly selected trees within three 10 m x 10 m plots were sampled. The chemical soil properties of the sites O-I and Y-I correspond approximately to ravine forest (RF) and grazing meadow (GM), sites O-II to spruce forest (SF) and Y-II to wet meadow (WM), sites O-III to spruce forest (SF) and Y-III to hay meadow (HM) according to the designations used by Garnica *et al*. [1]. Similarly, main climatic variables are given in Garnica *et al*. [1]. Deeper soil horizons however differ significantly in their origin and chemical properties: at 900 m it is composed mainly by dolomite; at 1300 m by the Kössen Formation; and 1500 m by the Allgäu Formation [28,35,36]. Precipitation and temperature are assumed to not differ significantly along the 600 m length elevation gradient.

During the 2015 growing season (October 13.), we collected fine roots and rhizosphere along an altitudinal gradient (elevations I, II and III) of a total of 90 Norway spruce (*Picea abies* (L.) H. Karst.) trees comprised half of young naturally regenerated [individuals below 2 m growing at the forest margin around ten-years-old (Y)] and half of mature artificially regenerated [fully grown individuals over 70-years-old (O)] trees. At each elevation, our sampling included 3 replicates (labelled 1, 2 and 3) for both ages (S1 Fig). Each biological replicate consisted of root material and rhizosphere from 5 trees that were pooled together.

For each tree, approximately 3–5 fine roots with visible ECM root tips and surrounding rhizosphere were collected and pooled replicates were stored in plastic bags at -20°C until further processing. Each replicate sample was cleaned from coarse soil particles under tap water and vital ECM root tip material and adhering fine soil particles were harvested under a stereo microscope in the laboratory. The material was then dried at 50°C in a desiccator overnight for subsequent DNA extraction.

### DNA extraction, PCR and Illumina sequencing

From each previously homogenized pooled sample, a subsample of 17.5mg of the dried material was used for DNA isolation with the innuPREP Plant DNA kit (Analytik Jena AG, Jena). For the identification of the fungal taxa, the internal transcribed spacer (ITS) region of the rRNA [37] was amplified using the fungal-specific primer ITS1F and ITS4 [38,39]. The MangoTaq (Bioline GmbH, Luckenwalde) polymerase kit was used for amplifying fungal DNA in a touchdown PCR [40]. The following conditions were employed for the PCR: 3 min at 94°C, 10 cycles of 30 s at 94°C, 45 s at 60°C (decrease by 1°C in each cycle) and 1 min 15 s at 72°C, followed by 26 cycles of 30 s at 94°C, 45 s at 50°C and 1min 15 s at 72°C. Final elongation was done for 7 min at 72°C.

PCR amplicons were normalized and incubated at 55°C for 5 min with the Nextera Amplicon Tagment Mix [41,42]. Libraries were subsequently amplified and index primers needed for the multiplexing of samples were added. The indexed libraries were cleaned up and size-selected (i.e. removing short library fragments) using magnetic AMPure XP beads, normalized and pooled in equimolar proportions. The pooled libraries of the fungal communities were then sequenced on an Illumina MiSeq instrument at the core facility c.ATG of the University of Tübingen.

### Quality control, processing and chimera detection of reads

The sequenced products for all samples were paired-end reads with a varying number of overlapping nucleotides between corresponding forward and reverse reads. Several preprocessing steps were necessary such as adapter clipping, merging of corresponding paired-end reads in the overlapping regions and finally low base quality trimming of the resulting reads [43]. After adapter removal, merging and trimming, the quality and distribution of read lengths of resulting reads were assessed with FastQC [44]. To identify possible contamination of Norway spruce DNA (other contaminations, e.g. from other plants and prokaryotes were not considered as we specifically selected roots containing rhizosphere from Norway spruce and used eukaryote specific PCR primers), reads were mapped to the host genome sequence [45] with the short-read aligner BWA-MEM [46], using a seed length of 40bp.

Since it has been shown that the sequencing of PCR amplicons can produce up to 23% chimeric reads [47], we checked for chimeric sequences with the UCHIME algorithm implemented in USEARCH v8.1 [48,49]. The algorithm takes a database of sequences that are known to be non-chimeric and calculates a score for the probability of each unique sequence to be chimeric. Here we used the fungal ITS reference sequences from NCBI’s BioProject PRJNA177353 [50] as references for UCHIME. The database currently contains 3709 fungal reference sequences (retrieved 19.05.2016). In order to reduce computational cost, identical reads were removed using the—derep_fulllength option of USEARCH prior to the application of UCHIME.

### Taxonomic classification and fungal diversity

To assess taxonomic affiliations of the query reads, these were aligned to the reference sequences in NCBI’s ITS RefSeq database using the MEGAN alignment tool (MALT) [29]. MALT was designed as an extension of the Metagenome Analyzer (MEGAN) [30] but can also be used independently. It uses a seed-and-extend approach similar to the BLAST algorithm [51] to find significant matches between query and reference sequences. By default, it employs spaced seeds to increase sensitivity [52,53] and a banded alignment algorithm that can perform both local and semi-global alignments [54]. MALT is able to assign a taxonomy based on the annotation of reference sequences along the NCBI taxonomy tree using the weighted LCA (wLCA) algorithm [55]. The weighted LCA algorithm assigns each read to the “lowest common ancestor” node in the taxonomy and thus provides a rather conservative strategy of assigning species or higher-level taxon labels. This results in a relatively high confidence of assignment to a higher taxonomic rank (i.e. to a genus, family or order depending on the taxonomic group). Version 0.3.6 of MALT was employed with several adjusted parameters (-wLCA -b 100—maxAlignmentsPerQuery 15—topPercent 2).

After combining samples in MEGAN, we exported assignments of reads to taxonomic labels at species and order levels for further analyses. As the reads were dereplicated in order to fasten up analyses, especially during the chimera detection, the counts per taxon were expanded (i.e. if an assigned read actually represented more than one read, we changed the count accordingly) again to get a less biased estimation of taxon abundances and thus community composition. The counts were normalized by the sample with the lowest total read count and the percentage abundance was calculated. The abundances for all fungal orders with an abundance over 1% in at least one sample and for all sample groups (average over replicates) were then plotted in a stacked barchart in order to visually investigate which fungal lineages are dominant components of the communities.

We identified the dominant predicted taxa in each sample by sorting all detected taxa and selecting those taxa that represent the upper 30% of read counts per sample. We then manually checked those species labels for plausibility (e.g. if the described native range is compatible with the study area).

We used the normalized counts on the species level to calculate Hill numbers [56] for all samples. Hill numbers are a general measure of biodiversity, defined as a reciprocal mean proportional abundance and differently weighing taxa based on their abundance, i.e. with different weighting of rare and abundant species or taxa. Hill numbers can be computed with different values for the order of diversity *q*, where values of *q<1* favour rare taxa (and *q = 0* simply corresponds to taxon richness), and *q>1* give more weight to common species or taxa. They also encompass other common indices of diversity such as Shannon (q = 1 is equivalent to the exponential of the Shannon index), Simpson (q = 2 is equivalent to the inverse of the Simpson index), etc.

The function 'specaccum' and the 'random' method from the R package 'vegan' were used to repeatedly add sites in a random order to the species pool and thus compute the species accumulation curve [57]. The size of the species pool for the complete sampling region was estimated with Chao's estimator implemented in the 'specpool' function of 'vegan' [57,58].

### Ecological characterization

Beside the taxonomic classification, the lifestyle of a fungal taxon and interactions of different lifestyles in a community are important aspects of fungal ecology. All taxa were therefore classified into ecological guilds with the FUNGuild tool and database (the database is available at www.stbates.org/funguild_db.php) [34]. The database currently consists of almost 10,000 records, and the classification is done predominantly on the level of genera.

### Statistical analysis

As a previous investigation by our group [1] indicated that the organic horizon does not differ significantly in its chemical properties for all sampling plots, we did not incorporate this in the analyses carried out in the present study. Since we sampled in a monoculture forest and in a distance from any other ECM plant species an influence of co-occurring vegetation can be discarded. For these reasons, we assessed the statistical significance of the variables altitude and host age by setting up a multivariate generalized mixed model (mGLM) and applying an ANOVA to the fitted model [59,60]. Two sets of read counts were used as multivariate data, one containing all detected taxa and one containing only those taxa that represented the topmost 30% of read counts (i.e. only the most abundant ones) in each sample.

Additionally, we visualized the relationships between taxon composition of samples and the environmental variables age and altitude by non-metric multidimensional scaling (NMDS) based on Bray-Curtis distances in the vegan package in R [57]. Environmental variables were fitted using the ‘envfit’ function of vegan. Significance of the variables were assessed with 999 random permutations.

## Results

### Illumina paired end sequencing

We recovered an average of 759,418 paired-end reads with a length of 2x241 bp from each pooled sample (ranging from 603,625 to 1,078,260 reads per pooled sample, see S1 Table). One million reads (approx. 4% of the total number of reads) were removed because they were too short or only contained adapter sequence. In total 65% of the paired-end reads with an average overlap of 125 bp could be merged and the remaining reads were analysed separately as unmerged single reads. Both variable parts of the ITS region were amplified and sequenced for the analyses in this study. However, as the Illumina Nextera protocol allows users to sequence fragments of the PCR amplicons of maximal 300 bp length sequences, most sequencing reads did not span more than one of the two variable spacers. About 5% of the reads could be mapped to the host genome of *Picea abies*, but hits were exclusively to a single 235bp contig marked ‘putative_contaminant’ (i.e. of presumably fungal origin) or to very short and low complexity regions (https://doi.org/10.6084/m9.figshare.6973781.v1). All reads were therefore kept for further analyses. In our study, after deduplication of the merged reads, the average number of reads per sample was reduced to 568,741. Applying UCHIME to the deduplicated reads, a total of 205,696 (2% of the total reads after quality filtering and merging) were identified as chimeric and were excluded from further analyses.

### Taxonomic coverage and belowground fungal community diversity

**Fig 2:**
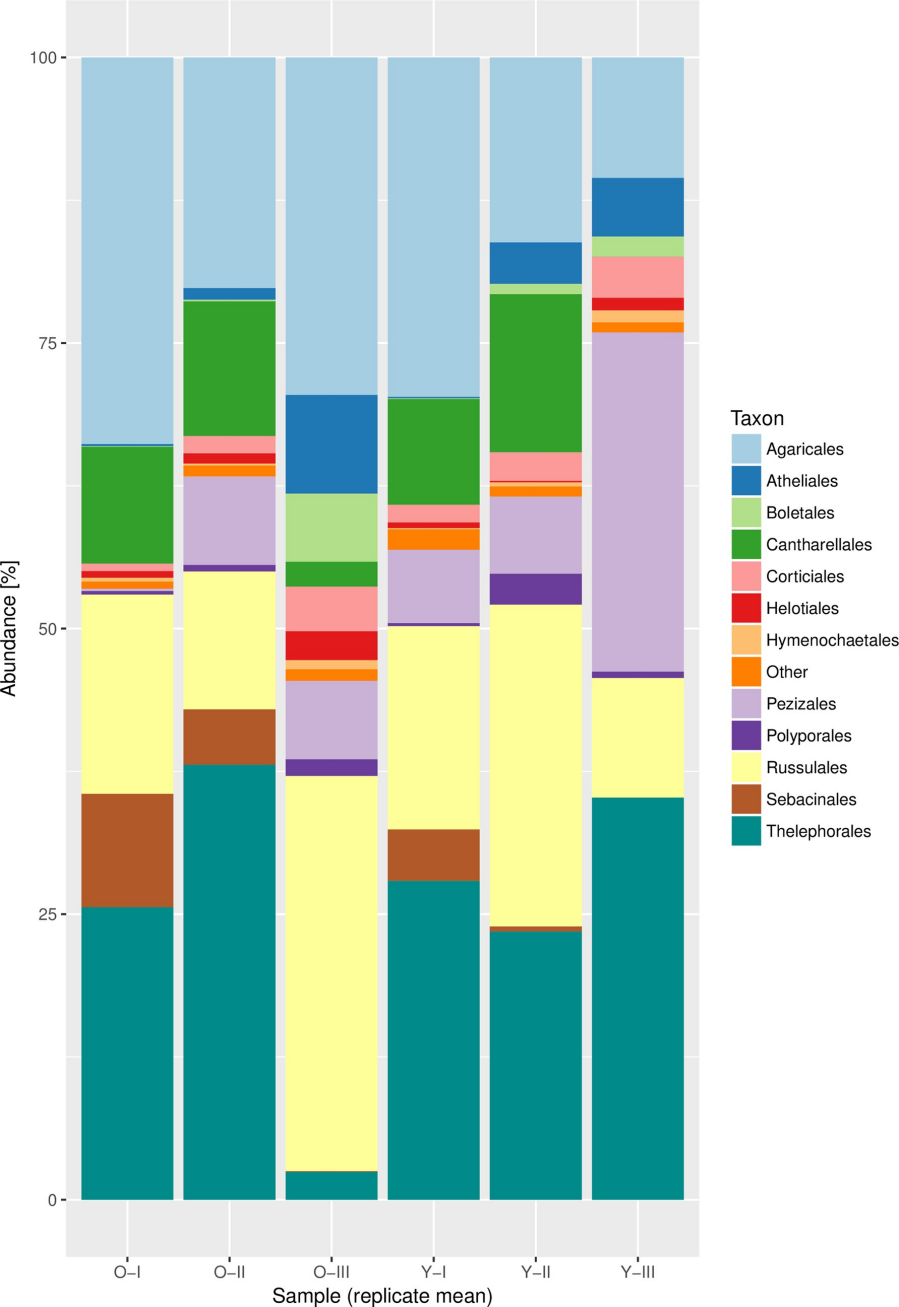
Abundances from normalized read counts for fungal orders detected in roots and surrounding soil from Norway spruce in the Bavarian Alps. Only orders with abundance >1% in at least one sample are shown, other orders are summed under ‘Other’. Sample replicate average is shown and labelled according to the age of the host tree ('O' for old, 'Y' for young individuals) and the altitude ('I','II','III', see Fig 1). See S2 Fig for all samples.

In total, 1285 fungal taxa on the species level distributed over 96 orders representing the phyla *Ascomycota* (11%) and *Basidiomycota* (89%) and *Chytridiomycota*, *Glomeromycota* and *Mucoromycota* (each <0.1%) were found. Only 0.03% of the reads were classified as taxa that were only identified from a single read (analogous to a ‘singleton’ in MOTU based studies). Richness of the pooled samples ranged from 167 to 506 (see S1 Table). The *Agaricales*, *Atheliales*, *Cantharellales*, *Russulales*, *Sebacinales* and *Thelephorales* from the *Basidiomycota* and the *Pezizales* from the *Ascomycota* represented the most abundant orders (Fig 2 and S2 Fig), all comprising at least partly ECM species. However, we also sequenced several saprophytic fungal taxa belonging to the orders *Corticiales*, *Hymenochaetales* and *Polyporales*, which where common across the sampled sites. Table 1 contains all taxa that represented the topmost 30% of read counts of at least one pooled sample (see S2 Table for the full list of detected taxa).

**Table 1:** Most abundant taxa in belowground fungal communities associated with Norway spruce.

Species	Provenance	O-I-1	O-I-2	O-I-3	O-II-1	O-II-2	O-II-3	O-III-1	O-III-2	O-III-3	Y-I-1	Y-I-2	Y-I-3	Y-II-1	Y-II-2	Y-II-3	Y-III-1	Y-III-2	Y-III-3
Chaetospermum chaetosporum	Europe/Asia	47 292	169	26 214	8 637	19 948	6 040	586	2	3	6 021	27 564	141	3 243	15	3	4	5	0
Tomentella parmastoana	Mahe	42 134	40	2 991	32 544	14 975	370	2	353	1 586	1 611	11 918	6 847	2 499	7 297	863	5 079	3	52 202
Cortinarius subtortus	Europe/North America	308	49 599	2	0	0	0	0	0	0	3	0	267	0	0	0	0	0	0
Lactarius austrotorminosus	Thailand	74	31 176	1 019	2 835	2 551	10 357	30 824	408	8 506	24	471	10 131	632	6 139	5 480	327	200	3
Hydnum vesterholtii	Spain/Europe	3	0	27 220	32 592	12	24	1	0	0	27	1 605	1 698	95 069	0	14	0	0	0
Inocybe lanatopurpurea	Europe	457	0	16 698	2	20 014	0	3	8	7	504	4 584	14 392	1 150	0	0	6	0	0
Craterellus indicus	India	479	10 279	14 035	218	0	0	0	0	1	0	0	32 690	93	0	0	0	0	0
Tomentella afrostuposa	West Africa	3 401	5 297	310	38 756	4 340	15 957	300	235	1 836	2 111	1 637	163	2 194	31 252	217	2 789	4	855
Hydnangium kanuka	New Zealand	17	0	35	34 886	3	0	1	37	0	5	4	7	7	0	0	1	0	0
Tomentella alpina	Austria	11 243	9 234	416	6 367	33 046	11 496	88	81	2 050	24 393	39 627	478	1 965	20 882	5 269	43 201	0	463
Tomentella hjortstamiana	Mahe	30	3	606	5 947	23 358	543	10	271	3	1 774	8 998	8	10	1 213	586	69	0	21
Tricharina praecox	Europe	24	185	0	3	19	55 424	4 802	35 246	6 657	38 174	460	927	144	2 566	45 118	17 096	183 158	8 519
Clavulina cinereoglebosa	Guaiana Shield	108	0	370	1 061	2	26 203	10 511	0	0	591	170	2	3 217	0	0	1	0	0
Russula jilinensis	China	9 243	3 198	13 663	2 415	6 824	627	35 054	2 323	15 805	0	634	18	2 553	2 862	24	13 671	9 377	0
Multifurca stenophylla	Australia	1 837	5 257	32	1 168	316	19	29 879	226	77	8	2	48	462	251	55	2 745	617	1
Gymnomyces xerophilus	USA	58	18	82	454	18 562	10 737	21 282	49 626	18 149	0	329	0	2 480	300	0	405	2 263	0
Xeromphalina setulipes	Spain	0	2	0	293	914	4 533	9 314	28 522	25 852	3	0	0	2 941	6 357	0	7 157	6 171	27 944
Cortinarius luteo-ornatus	Europe	0	0	3	6	0	0	3	12	23 803	0	17	0	12	10	0	0	0	7
Xerocomellus sarnarii	Spain/Europe	6	0	0	2	0	6	116	82	22 017	0	0	2	3	10	0	114	0	8
Lactarius horakii	Asia	6 232	7 497	608	209	129	167	13 999	14	121	41 807	485	202	360	42 385	54 903	16 603	8 578	123
Lactarius alboscrobiculatus	Thailand	2 356	1 590	2 895	6 839	6 508	2 977	512	12	30	9 691	463	64 584	11	18 430	40 810	5 174	2 413	33
Tomentella agbassaensis	Benin (West Africa)	15 920	2 220	2 102	15 848	7 134	1 364	4	96	809	2 195	10 806	2 543	1 237	4 004	4 222	33 983	0	37 041

These 22 taxa (species) represent the upper 30% of read counts for all samples. Note that many reference taxa (species) do not occur in the sampling area, indicating the defectiveness of the reference database. For many of these taxa (species), members of the same genus are known or very likely to occur in the Bavarian Alps.

**Fig 3:**
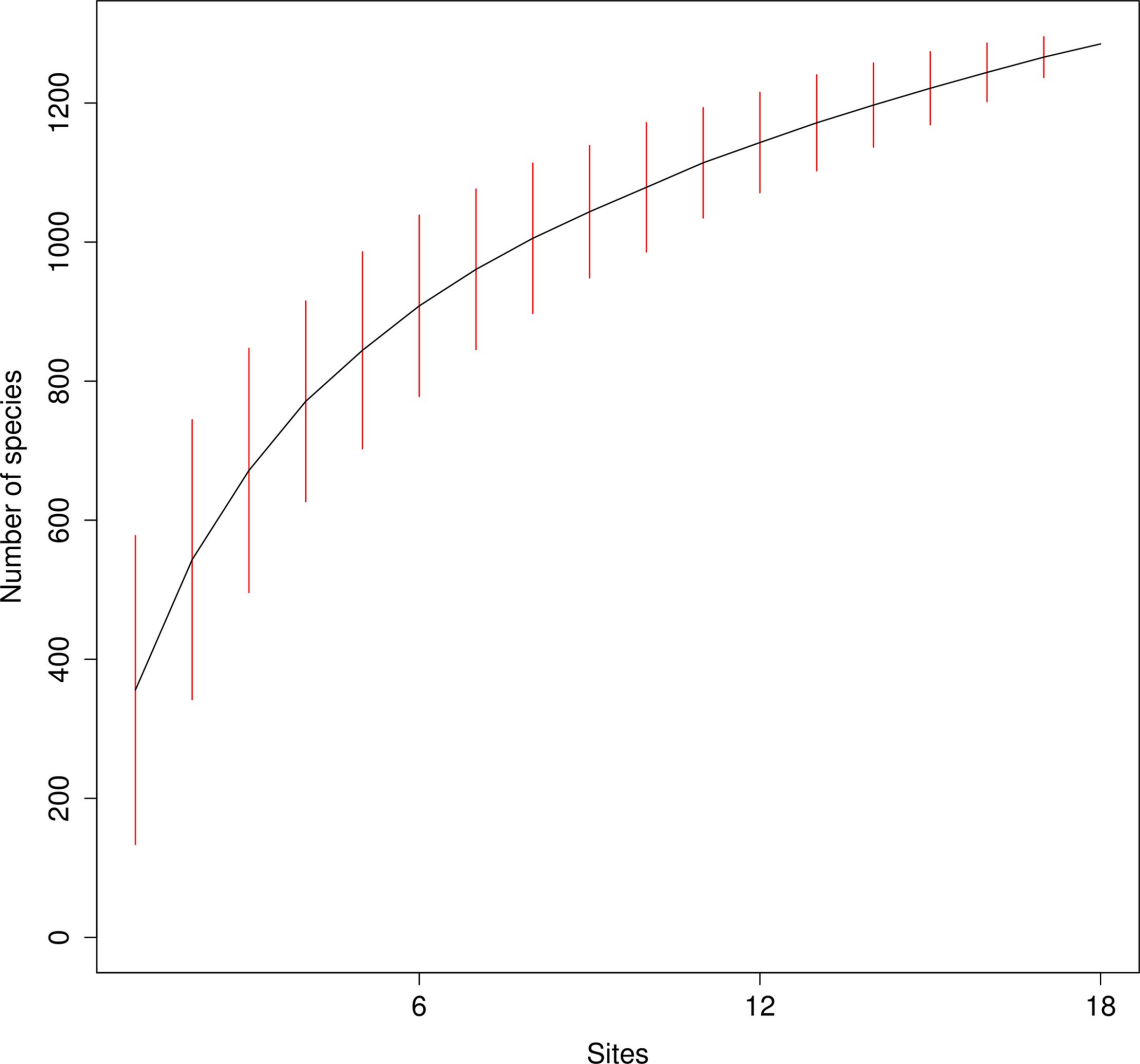
Taxa/species accumulation curve for fungal belowground communities associated with Norway spruce. The 'random' method from the 'specaccum' function implemented in the R package 'vegan' [57] was used. Confidence intervals are shown as red bars.

**Fig 4:**
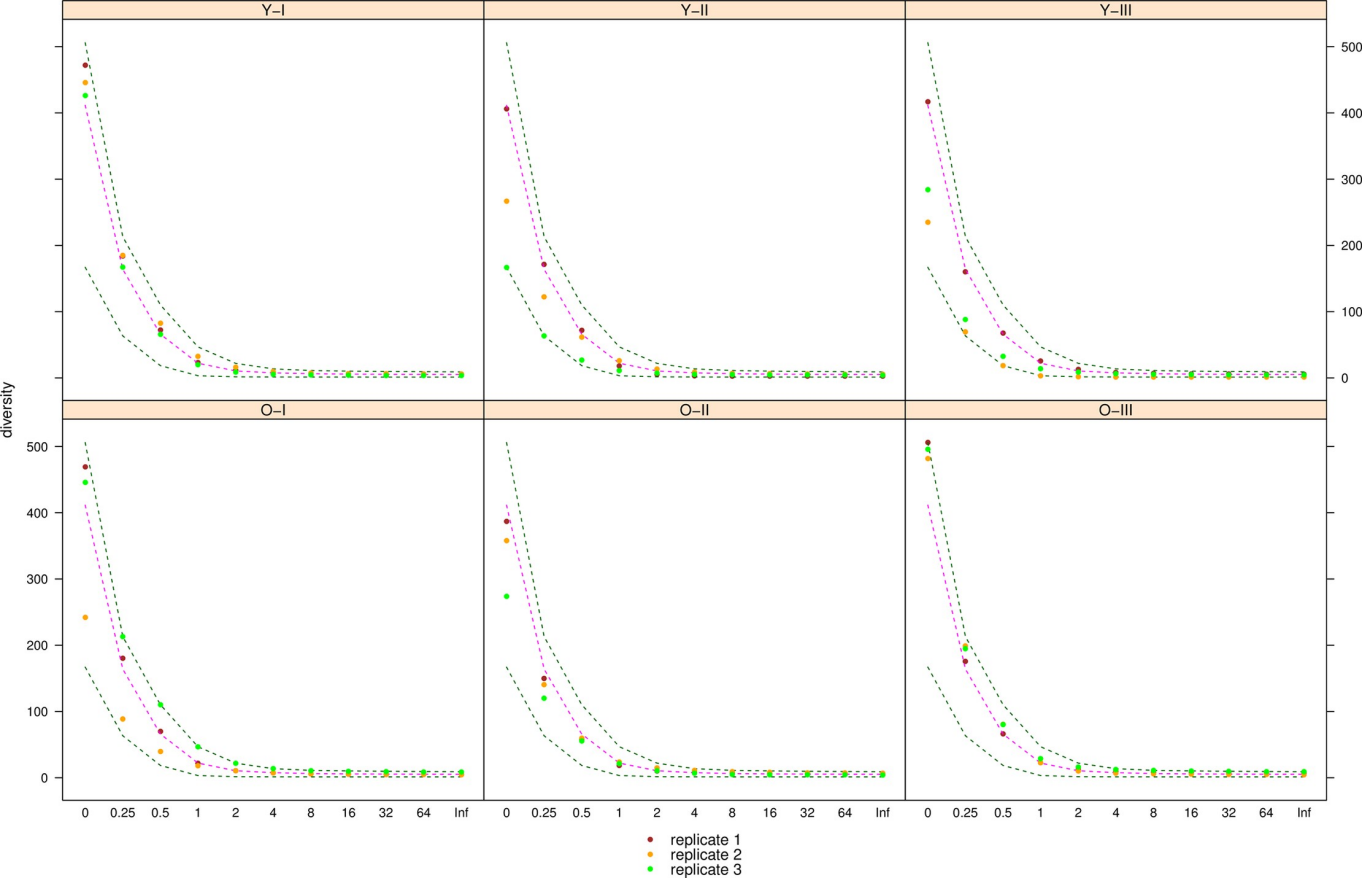
Alpha-diversity (circles) of belowground fungal communities associated with Norway spruce along an altitudinal gradient in the Bavarian Alps. Each sample is labelled according to the age of the host tree ('O' for old, 'Y' for young individuals), the altitude ('I','II','III', see Fig 1). The three replicates for each sample are plotted together in a panel with different colors ('1': red, '2': yellow, '3': green). For each sample, Hill numbers for different values of *q* (order of diversity) were calculated, giving different weights to species abundances. Green dotted lines show minimum and maximum across all samples, pink dotted lines show the median.

Despite the large number of taxa detected in each individual as well as the total of all sampled plots, the species accumulation curve showed no saturation, indicating that not all taxa present in the local species pool were sampled (Fig 3). The putative size of the species pool in the sampling area as calculated with Chao's estimator was 1650 with a standard error of 51, suggesting that approximately 400 taxa from the regional species pool might have been missed in the biodiversity survey. The diversity indices of the belowground fungal communities are illustrated in Fig 4. The highest taxon diversity was found in mature trees at both lower and upper plots (O-I-3 and O-III-3), whereas the lowest diversity was found in young trees at medial and upper plots (Y-II-3 and Y-III-2).

### Relationships between belowground fungal communities and altitude

The statistical test of the fitted mGLM model reported a significant effect of altitude (*p*<0.005) on the composition of both the whole fungal communities as well as the most abundant part of the fungal communities, but not on the fungal taxon diversity (Table 2).

**Table 2:** Effect of host plant age and altitude on belowground fungal communities associated with Norway spruce.

	Composition all		Composition 30%		Diversity (0)		Diversity (0.25)		Diversity (0.5)		Diversity (1)		Diversity (2)	
	Dev	p	Dev	p	Dev	p	Dev	p	Dev	p	Dev	p	Dev	p
Altitude	6001.731	**0.001**	178.09	**0.003**	3.939	0.155	2.247	0.311	2.053	0.371	2.676	0.3	1.798	0.437
Age	2298.454	**0.047**	47.58	0.072	1.886	0.221	1.934	0.202	2.063	0.185	2.226	0.162	3.431	0.083
Altitude*age	2957.317	**0.005**	87.46	0.170	5.132	0.142	4.653	0.18	3.515	0.273	1.422	0.587	0.851	0.712
			diversity (4)		diversity (8)		diversity (16)		diversity (32)		diversity (64)		diversity (Inf)	
			Dev	p	Dev	p	Dev	p	Dev	p	Dev	p	Dev	p
Altitude			0.853	0.643	0.131	0.929	0.271	0.881	0.267	0.836	0.158	0.887	0.023	0.984
Age			5.109	**0.016**	4.983	**0.023**	4.583	**0.024**	4.482	**0.018**	3.708	**0.033**	3.795	**0.025**
Altitude*age			0.959	0.553	1.238	0.478	0.835	0.522	0.713	0.552	0.718	0.574	0.651	0.581

The multivariate generalized linear model was fitted with the 'manyglm' function in the R package 'mvabund' [59]. Significance was calculated in an ANOVA, statistically significant p-values are highlighted in bold. The influence of age and altitude on the species abundances and the diversity (Hill numbers ^q^D) of orders 0 to Inf was computed. Besides the p-value, the deviance (Dev) of each model is given as a measure of the quality-of-fit.

**Fig 5:**
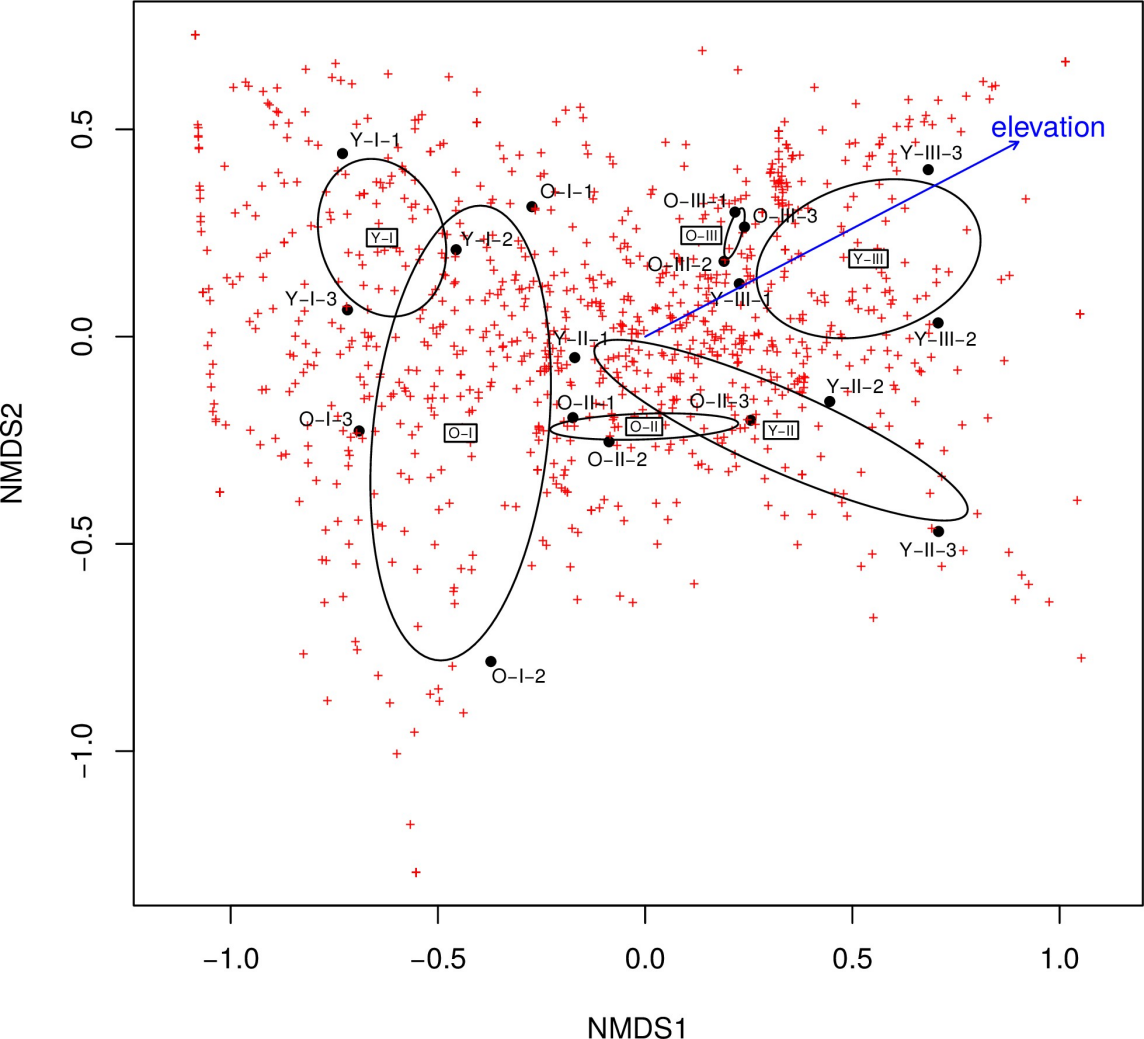
NMDS ordination plot of all sampled sites, based on the composition of fungal taxa (on the level of species, stress = 1.4). All explanatory variables that were found to have a significant influence (*p*<0.05) are shown as vectors. Each sample is labelled according to the age of the host tree ('O' for old, 'Y' for young individuals), the altitude ('I','II','III', see Fig 1) and the replicate number ('1','2','3'). For the 6 biological replicate groups (Y-I to O-III), ellipses are drawn according to the standard deviation of point scores (function ordiellipse in vegan).

Similarly, the NMDS plot (Fig 5) shows a clustering of samples from the same altitude (stress = 0.14), and the *p*-value for the significance of the altitude on fungal taxon composition was 0.001.

### Relationships between belowground fungal communities and plant age

The statistical test of the fitted mGLM model reported a significant effect of host age (*p*<0.05) on the diversity of the fungal communities for q>2, so when down-weighing rare taxa (Table 2). The composition of fungal taxa in general, but not of the most abundant taxa of the communities was also significantly influenced by plant age (p<0.05). We also assessed statistical significance of the plant age on fungal taxon composition but found this to be not significant (*p* = 0.57).

### Ecological guilds of sampled taxa

**Fig 6:**
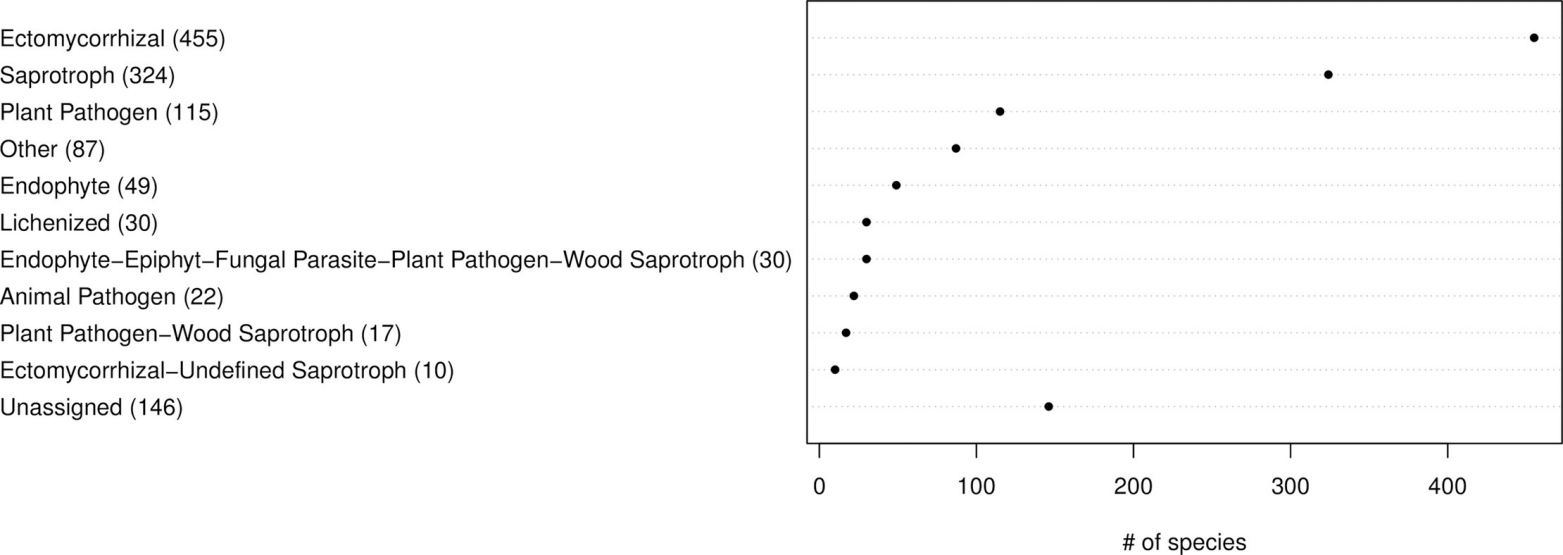
Ecological guilds of fungal taxa in belowground communities associated with Norway spruce. The taxa were classified into 47 ecological guilds with the FUNGuild tool and database [34]. For ease of reading, all guilds containing less than 10 taxa were grouped together as ‘Other’. See S2 Table for a complete list of assigned guilds.

The fungal taxa were grouped into 31 ecological guilds; the most diverse guilds were ECM with 389 taxa, saprotrophs with 195 and plant pathogens with 60 taxa (Fig 6).

## Discussion

### Taxonomic coverage and belowground fungal community diversity

With 1,285 detected fungal taxa at approximately the species level, fungal diversity detected in our study is relatively high compared with that found in a subtropical forest [61], and herbaceous plants from alpine and arctic regions using 454 sequencing [62–64]. However, these comparisons should be interpreted very carefully considering differences in type of samples, sample processing and applied molecular methodology. The species that are absent from the local species pool (400 out of 1650, see also Fig 3) could be members of fungal groups that were not detected with the primer set we used [65].

The dominant orders detected here (Fig 2 and S2 Fig) were also reported as abundant in previous studies from other ECM plants and ecosystems [63,66,67]. Similarly, various taxa previously reported from fruiting body surveys in the Bavarian Alps and adjacent regions were also identified by our Illumina MiSeq sequencing (see S2 Table). Among the most common taxa (see Table 1) were *Tomentella alpina* recently described by Peintner and Dämmrich [68] from the Tyrolean Alps and *Cortinarius subtortus* that is a typical ECM species growing between *Sphagnum* in the Bavarian Alps. On the other hand, several of the abundant taxa are characterized by conspicuous fruiting bodies, which, based on our basidiomata/ascomata surveys and literature do not occur in the study area. This discrepancy is very likely due to the fact that the reference barcode database used to assess the taxon diversity (the NCBI ITS RefSeq in this case) is incomplete and a match to the nearest related species present is found instead of the 'correct' species label. Some erroneous matches correspond to the Asian species *Lactarius alboscrobiculatus*, the Thai species *Lactarius austrotorminosus* and the Guyanese species *Craterellus strigosus*, which should represent species that are commonly collected in the study area: *Lactarius scrobiculatus*, *L*. *torminosus* and *Cantharellus lutescens*, respectively.

Moreover, a comparison of our study with previous field surveys of our working group allowed to detect a major number of taxa lacking or developing inconspicuous fruit bodies especially from several orders in the *Basidiomycota* (e.g., *Atheliales*, *Thelephorales*, *Sebacinales*) and *Ascomycota* (e.g., *Helotiales*, *Pezizales*) (Fig 2).

### Relationships between belowground fungal communities and altitude

The effect of altitude on the composition of the most abundant members of the fungal communities (see Fig 5) suggests that an unresolved combination of several environmental conditions produce very distinct fungal communities. Competition might however also play an important role in shaping the species composition. Most likely an interaction of both biotic and abiotic factors is controlling which fungi thrive where. In addition, it seems that certain fungi are affected more by these factors than others. Several taxa displayed restricted distributions along the gradient (see Table 1), e.g. *Cortinarius subtortus* was only common at the lower altitudes whereas *Zelleromyces hispanicus* was significantly more frequent mid altitudes, findings that agree with previous sporocarp surveys (Garnica, 2016, pers. comm.). The low presence or absence of members of the order Sebacinales at the highest plots of the altitudinal gradient (see Fig 2 and S2 Fig) can be explained by the fact that both *Sebacina epigaea* and the *S*. *inscrustans*-complex prefer mineral soil lacking grasses, which is not the case in these sites (see Fig 1). In contrast, the *Byssocorticium caeruleum* and the likely mycorrhizal sebacinoid *Chaetospermum chaetosporum* were found at all altitudes and also independently of the plant host age. Similar results have been reported for fungal communities growing on common woody substrates [17], in the phyllosphere of European beech (*Fagus sylvatica*) [20], in a community of ericoid mycorrhizae and root-associated fungi of *Vaccinum membranaceum* [22], and for ectomycorrhizal fungi of *Pinus sylvestris* [23], along elevational gradients of 1900 m, 1000 m, 1000 m and 300 m, respectively. A comparison of these studies with our work suggests a similar effect of elevational gradient (the relative change in elevation) and associated abiotic factors on the composition of diverse and ecologically different communities of microorganisms, whereas the diversity was affected little (i.e. no significant difference in diversity over the gradient, see Fig 4).

We suggest that the similarity between patterns of fungal diversity detected (i.e., no significant change in diversity) in Meier et al. [17], who analysed a gradient of 1900 m, and this study, is due to the relative homogeneity of host plants (monoculture of *Picea abies* with nearly constant soil properties) and substrate (wood) respectively along both transects. This result is in contrast to our account of taxon diversity based on sporocarp surveys (Garnica, 2016, pers. comm.) as well as earlier studies by Kernaghan & Harper [11] and Bahram et al. [14] for ectomycorrhizal fungi, and Wu et al. [12] and Lugo et al. [13] for arbuscular mycorrhizal fungi, where species diversity decreased as altitude increased. Similarly, our results differ from those of Miyamoto et al. [15] and Meng et al. [18], who detected the highest diversity of ectomycorrhizal fungi and bacteria, respectively, at mid elevations. Furthermore, Egan et al [10] reported a lower diversity of arbuscular mycorrhizal fungi in alpine communities relative to lower elevation communities. Siles et al. [19] found that changes in fungal diversity were mainly influenced by pH and C/N and Cordier et al. [20] postulated that temperature was a main cause of the variation in species composition in their phyllosphere studies of European beech.

Kivlin et al. [69] in a recent meta-study on elevational patterns in fungi, also came to the conclusion that the patterns of fungal diversity ‘were not influenced by the elevation range sampled’. When comparing the results discussed above there seems to be no clear connection between the pattern of diversity and the length of the elevational gradient. None-the-less it remains somewhat unclear as to how these different patterns develop, indicating that further research in this topic is clearly needed.

### Relationships between belowground fungal communities and plant age

The diversity associated with young trees is limited both in terms of fungi that are ‘available’ in the soil (as they are not big and old enough to create their own microhabitat) as well as by the small root system that only provides limited space for mycorrhizal interactions. This also corresponds to previously reported patterns of fungal diversity in relation to plant age [70]. The similarities of fungal community composition between young and mature individuals of Norway spruce could suggest that the old trees can act as reservoirs of fungal inoculum for naturally regenerated trees [71], as young trees share up to 82% of taxa with their neighbouring mature forest trees (see S1 Table).

### Ecological guilds of sampled taxa

In contrast to studies based on morphological features, which encompass mostly those conspicuous species, next-generation sequencing allows us to detect taxa developing very ephemeral and/or inconspicuous fruiting bodies or lacking these. Although the approach employed here is rather focused on ECM species characterization, it does not neglect other fungal taxa with different ecology present in the most proximal rhizosphere. It is hence comparable to the approach that Song *et al*. [72] proposed for the sampling of soil fungal communities. The high frequency of the saprophytic taxa detected in our study is presumably also linked to the fact that we did not use sterilized root tip surfaces.

## Conclusions

Our findings suggest that altitude and plant age affect the composition and diversity of the belowground fungal communities in the fine ECM roots and rhizosphere of Norway spruce. Using a sampling strategy that captures all types of fungi living in close proximity to roots and rhizosphere, we detected more diverse and distinct belowground fungal communities compared to those known from fruiting body surveys. This study also validates bacterial microbiome techniques for a fast and accurate characterization of the diversity and composition of fungal communities in the framework of DNA metabarcoding studies. However, through an expanded fungal reference sequence database, the usefulness of metabarcoding studies for biodiversity monitoring and conservation studies in changing ecosystems of this ecological relevant group of microorganisms would increase significantly. Enhancing the local and global reference sequences of correctly barcoded species should thus have the highest priority for biodiversity researchers and taxonomic specialists, preferably in a united effort such as the ITS RefSeq Project.

## Supporting information

S1 Table Samples of belowground fungal communities associated with Norway spruce. Identifiers are given according to plant host age, altitude and replicate number (see S1 Fig). For each sample, the number of sequenced paired-end reads and the number of species and orders detected (against the NCBI ITS RefSeq) are presented. Additionally, the number and percentage of overlapping species between two samples of the same plot (old and young trees) are included (and denoted as ‘overlap to neighbour’). Note that the number of raw sequences is given as the number of read pairs, while the number of clipped and merged reads is given as single reads.
(XLSX)

S2 Table All species that were detected in this study. The normalized counts per sample as well as the overall percentage abundance are given as well as the corresponding ecological guilds and trophic modes as predicted by FUNGuild and those used in Fig 6.
(XLSX)

S1 Fig Location of the 9 sampling sites in the study area. Samples were taken at three different altitudes along an altitudinal gradient from 900 to 1500 m a.s.l. (I: 900 m, II: 1200 m, III:1500 m a.s.l) in the Bavarian Alps (north side of Iseler Mountain, Germany). At each location samples from old and young individuals of Norway spruce were sampled.
(PDF)

S2 Fig Abundances from normalized read counts for fungal orders detected in roots and surrounding soil from Norway spruce in the Bavarian Alps. Only orders with abundance >1% in at least one sample are shown, other orders are summed under ‘Other’. Samples are labelled according to the age of the host tree ('O' for old, 'Y' for young individuals), the altitude ('I','II','III', see Fig 1) and the replicate number ('1','2','3').
(PDF)
