# Supplementary figures and images for: Belowground fungal community diversity and composition associated with Norway spruce along an altitudinal gradient
Source: PLoS One. 2018 Dec 5;13(12):e0208493. doi: 10.1371/journal.pone.0208493 (PMC6281267; doi:10.1371/journal.pone.0208493)

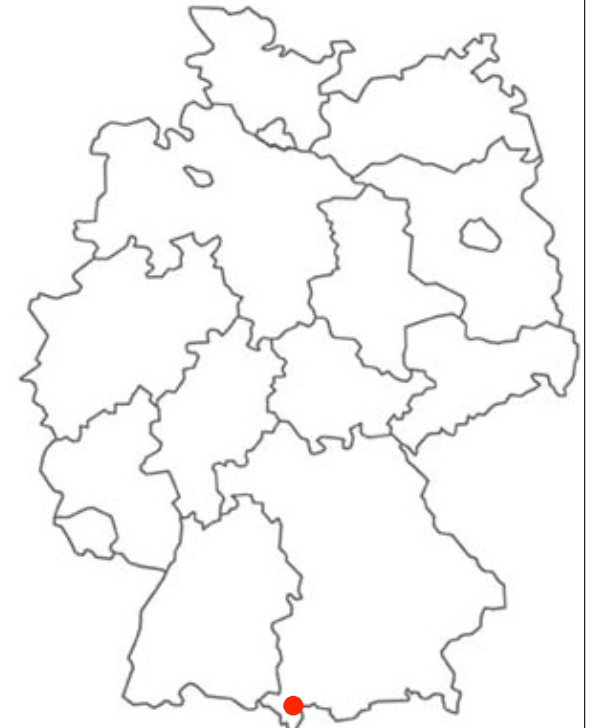

©OpenStreetMap contributors  
([openstreetmap.org](https://openstreetmap.org)),  
OpenTopoMap (CC-BY-SA)  
1:15000

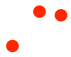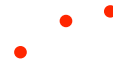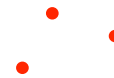

Supplement: S1 Fig — Samples were taken at three different altitudes along an altitudinal gradient from 900 to 1500 m a.s.l. (I: 900 m, II: 1200 m, III:1500 m a.s.l) in the Bavarian Alps (north side of Iseler Mountain, Germany). At each location samples from old and young individuals of Norway spruce were sampled. (PDF) [file pone.0208493.s003.pdf]

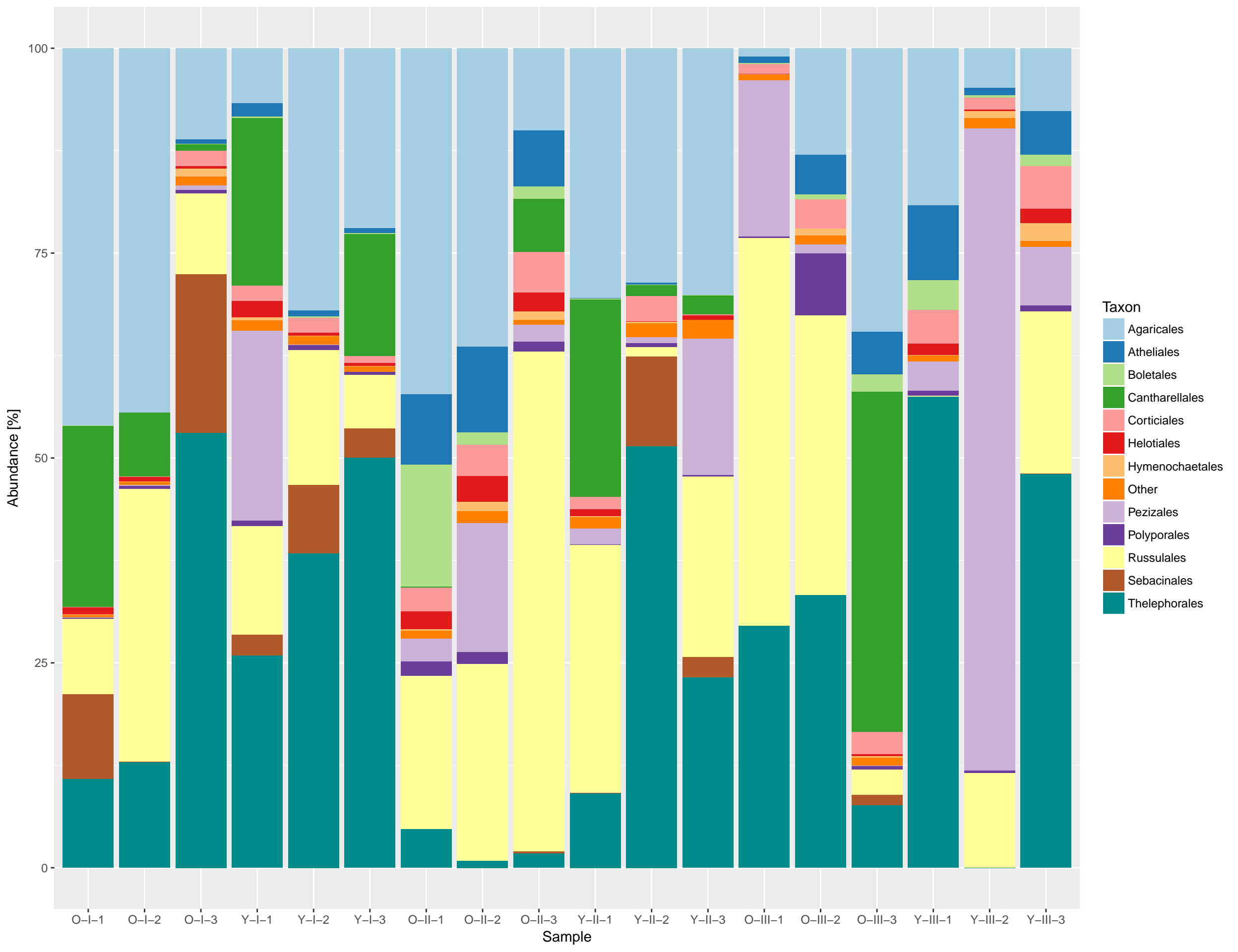

Supplement: S2 Fig — Only orders with abundance >1% in at least one sample are shown, other orders are summed under ‘Other’. Samples are labelled according to the age of the host tree ('O' for old, 'Y' for young individuals), the altitude ('I','II','III', see Fig 1) and the replicate number ('1','2','3'). (PDF) [file pone.0208493.s004.pdf]
